# Nutritional Needs in Mental Healthcare: Study Protocol of a Prospective Analytic Observational Study Assessing Nutritional Status, Eating Behavior and Barriers to Healthy Eating in Psychiatric Inpatients and Outpatients Compared to Healthy Adults

**DOI:** 10.3389/fpsyt.2022.906234

**Published:** 2022-06-14

**Authors:** Florian Hotzy, Ladina Risch, Sonja Mötteli

**Affiliations:** ^1^Department of Psychiatry, Psychotherapy and Psychosomatics, Psychiatric Hospital of the University of Zurich, Zurich, Switzerland; ^2^Faculty of Medicine, University of Zurich, Zurich, Switzerland

**Keywords:** nutritional status, eating behavior, attitudes, nutrition knowledge and skills, mental disorder, severe mental illness, psychiatric treatment, inpatient

## Abstract

**Background:**

Mental disorders are often associated with unhealthy eating behaviors and metabolic comorbidities. This can result in reduced life expectancy and poorer quality of life in people with mental disorders. This study protocol describes an observational study that examines the nutritional status and eating behaviors of people with severe mental illness (SMI) and the need for psychiatric treatment who were between 18 and 65 years old. In addition, the study focuses on possible barriers for healthy eating that have not yet been examined in this population.

**Methods:**

A total of 192 study participants will be recruited: 64 inpatients and 64 outpatients from the Psychiatric Hospital of the University of Zurich with SMI as well as 64 healthy individuals from the general population as a control group. The participants will be interviewed regarding their nutritional status, eating behavior, nutrition knowledge, food and cooking skills, personality, attitudes and feelings toward nutrition. In addition, the severity of symptoms and several control variables (e.g., sociodemographic variables and physical activity) will be assessed. For the patient samples, data will comprise routine medical data, and, if available, routine laboratory data. Inpatients will be interviewed once at the beginning of their treatment. Outpatients will be interviewed at the beginning and after 3 months of treatment (same interview questions). Healthy adults of the control group will be interviewed once at any time during the recruitment period.

**Discussion:**

The described study will identify nutritional needs and possible barriers to healthy eating in patients with mental disorders. The results will help to define recommendations for nutritional risk screening in psychiatric patients and for planning effective nutritional interventions.

## Introduction

At least one in three individuals is affected by mental health problems during their lifetimes. People with mental illnesses, such as affective or psychotic disorders, have substantially reduced life expectancies compared to the general population, mainly due to poor physical health conditions (1).

A recent comprehensive report by the Lancet Psychiatry Commission showed that individuals with mental disorders have significantly higher rates of obesity, diabetes and metabolic syndrome with a 1.4-2-fold increased risk for cardiovascular and metabolic diseases compared to the general population (2). Along with the side-effects of psychotropic medications, the report revealed various “lifestyle factors,” such as inactivity, poor diet and smoking, as modifiable behavioral risk factors for physical comorbidities in people with mental disorders. For example, people living with severe mental illness (SMI), including major depression, schizophrenia and bipolar disorders, have a higher caloric intake and a lower diet quality that includes more pro-inflammatory foods (e.g., red and processed meats, sweets, French fries and refined grains) and less healthy foods (e.g., vegetables and fruits), compared to the general population (3, 4). Therefore, nutritional interventions in psychiatry might be promising given that a healthy diet can reduce inflammation (5) and obesity in people with SMI (6). In addition, the supplementation of some nutrients might also have positive effects on the course of the disease (7). Diet has suggested to be as important to the discipline of psychiatry as it is to cardiology, endocrinology, and gastroenterology (8).

The planning and implementation of effective nutritional interventions in mental healthcare require a valid assessment of patients' nutritional status, challenges and needs. However, to date, no validated nutritional risk screening methods that also consider overnutrition exist for people with mental disorders (9). Similar to the somatic hospital setting (10), regular nutritional risk screening should be established in mental healthcare. However, nutritional risk screening should not only focuses on the identification of undernourished or malnourished patients, but also include the assessment of the risk for overnutrition and other aspects, such as constipation, dysphagia or problematic eating behaviors (9, 11). Overlooking poor nutritional conditions can result in higher complication rates, higher mortality rates and longer hospital stays (10).

The limited availability of healthy food, inadequate social support, a low socio-economic status or a limited budget, along with taste preferences and daily habits, are frequently reported barriers to healthy eating not only in people with mental disorders (12–16), but also in the general population (17, 18). In addition, people living with SMI are often at risk for food insecurity due to financial problems and/or precarious housing situations (12, 13).

Moreover, individuals with mental disorders experience illness-related barriers, such as increased appetite caused by psychotropic medications (particularly antipsychotics such as olanzapine and clozapine), or reduced motivation and/or ability to process information due to cognitive impairments, which result in decreased shopping and cooking activities (12). They are also assumed to have less nutrition knowledge, and fewer food and cooking skills compared to the general population, but there is little research available on this topic (12, 14–16, 19). The degree of capacity to process and understand nutrition-related information as well as nutrition knowledge and skills necessary to make healthy food choices are summarized as “nutrition literacy,” which predicts adherence to healthy eating behaviors in people with diet-related illnesses such as dyslipidaemia, hypertension or being overweight (20).

In addition, personality traits might be another barrier to healthy eating behaviors in this population. It was shown that personality traits, such as conscientiousness, agreeableness, and extraversion, are linked to healthier eating behaviors, whereas neuroticism is linked to more unhealthy eating behaviors (21–23). High values for neuroticism have also been suggested to be a risk factor for the development of disordered eating behaviors (24).

Aside from personality traits, people also differ in the extent to which they perceive their personal characteristics, such as intelligence or mental health, as being more or less stable or changeable. Some theories describe these underlying perspectives. Entity theory describes attributes as relatively stable, and incremental theory describes attributes as more flexible and changeable (25, 26). The influence of implicit theories on attitudes and behaviors has been studied across many fields (27). Research has shown that people with incremental beliefs are more successful in goal setting and monitoring, for example, when it comes to weight management (28). It might be especially important for people with mental disorders to hold or develop incremental beliefs regarding mental and physical health outcomes, but to our knowledge, research on this topic is scarce. Incremental beliefs might also be beneficial in questioning or adjusting attitudes or emotions.

Healthy eating behaviors depend on many food-related decision-making situations during the day. Aside from attitudes, our decision-making is guided by emotions (29). Emotions are effective when fast decisions are needed, especially in complex situations or when mental resources are limited (29), which often characterizes daily food decisions. In particular, people with mental disorders seem to have difficulty making healthy food choices, as they might feel confused/ambivalent emotions due to the availability of various food options (14). Therefore, to foster healthy eating in people with mental disorders, it might be helpful to address people's feelings toward nutrition and to provide simple food decision strategies.

### Aims and Hypotheses

This paper presents the study protocol of an observational study that examines the nutritional status and eating behaviors of people with SMI and the need for psychiatric treatment along with determinants for healthy eating, such as nutrition knowledge, food and cooking skills, personality, attitudes and feelings toward nutrition, that have been less studied in this population. Based on the reported literature, we assume that people with mental disorders have, on average, a poorer nutritional status and more unhealthy eating behaviors. Probably, they might also have lower levels of nutrition knowledge and fewer food and cooking skills compared to healthy adults from the general population. We further examine whether higher levels of conscientiousness, incremental beliefs and positive feelings toward nutrition are associated with healthier eating behaviors and whether a reduction in psychiatric symptoms (based on psychiatric treatment) is related to improvements in eating behavior.

## Methods

### Design

This observational prospective cohort study involves three different samples: psychiatric inpatients, psychiatric outpatients and a control group of healthy adults. “Healthy” is defined as the absence of a mental disorder. Data will be collected by interviews and anthropometric measures. In the patient samples also routine medical data, and, if available, routine laboratory data will be used. Inpatients will be interviewed once at the beginning of their treatment. Outpatients will be interviewed at the beginning and after 3 months of their treatment (same interview questions) to investigate the relationship between psychiatric symptoms and eating behavior (which cannot be properly investigated in inpatients due to the consumption of prepared meals in the hospital). Healthy adults of the control group will be interviewed once at any time during the recruitment period.

### Participants

Our target sample is composed of patients with affective or psychotic disorders who need intensive inpatient or outpatient treatment at the Psychiatric Hospital of the University of Zurich (Psychiatrische Universitaetsklinik Zuerich, PUK). Based on previous research with a comparable patient population (30), we assumed that patients with affective or psychotic disorders who are treated at the inpatient and outpatient facilities of the PUK comply with the criteria for SMI regarding disability and the duration of illness (31).

#### Inclusion Criteria

- Only patient samples: Inpatient or outpatient treatment at the PUK.
- Only patient samples: Main (preliminary) psychiatric diagnosis classified as F2 or F3 according to the ICD-10.
- Age between 18 and 65 years.
- Sufficient communication skills in the German language.
- Permanent residence in the canton of Zurich or close neighboring areas.
- Ability to give written informed consent.
- Adequate and cooperative/able to participate in interviews.

#### Exclusion Criteria

- Other main psychiatric diagnoses.
- Diagnosed eating disorder (based on medical records and self-reports).
- Only healthy adults of the control group: Diagnosis or presence of a mental disorder (self-reports).

### Recruitment

Based on previous admissions to the Psychiatric Hospital of the University of Zurich, we expect to enroll 1–2 participants per week per setting, resulting in a recruitment time of up to 1 year. Recruitment of the participants started in September 2021.

Inpatient participants (*n* = 64) were/will be recruited from seven wards for the acute treatment of mental disorders, and outpatient participants (*n* = 64) were/will be recruited from the day clinic and ambulatory of the PUK at their admission. A list of all new patients in the respective settings will be screened on a daily basis by clinical staff members to identify eligible participants. They will consecutively invite potential participants and inform them about the study's purpose. If the patients agree to participate and give written informed consent, the clinicians will hand over the participants' contact dates to the study team.

In addition, healthy adults from the general population (*n* = 64) living in the canton of Zurich or close neighboring areas will be recruited as the control group in our study. We will use community-based recruitment strategies, such as advertisements and flyers posted in public places (e.g., working and shopping areas or sports and healthcare institutions). To obtain comparable groups, we will use stratified samples in relation to gender and age (see Sample size calculation). If the planned number of a subgroup (e.g., 16 male participants 18–40 years) will be achieved, individuals of this group are no longer eligible participants.

### Procedure

Study participants will be interviewed by the research assistants of this study. The interviews will take place at the PUK or at the place of choice in terms of the control group. Prior to the start of the study, the research assistants received a specific interview training including the standardized assessment of anthropometric measures. After 3 months they receive refresher session with a duration of 2 h to ensure correct data assessment and recording. The assessments, including measurements and interviews, will last an average of 1.5 h and can be interrupted or conducted in two or more sections according to the patients' preferences and mental conditions. The participants will receive a drink of their choice from the interviewer. They will not receive any financial compensation. All items of the assessments will be directly implemented in LimeSurvey (32), which is an appropriate online tool for research purposes. The participants' answers and data will be encoded, and no identifying data will be recorded in the online survey. For the inpatient and outpatient samples, the interview data will be completed with specific medical routine data of the patients.

### Outcome Measures

Table 1 displays the outcomes, predictors and control measures. Data will be assessed from measurements and interview data or drawn from the patients' medical records.

**Table 1:** Overview of measures.

	**Instrument/Measurement**	**Source of data**	**Sample**
**Nutritional status (outcomes)**		
Anthropometric measurements		
Body mass index (BM)	Kg/m2; weight and height assessed using digital scale SOEHNLE 63850 PWD Style Sense Compact 100 and SECA measuring tape	Interview	All
Waist to hip ratio (WHR)	Abdominal girth/hip girth assessed using SECA measuring tape	Interview	All
Nutritional risk scores		
Score 1	Nutritional risk screening (NRS) including modified Global Assessment of Functioning Scale (m-GAF)	Interview	All
Score 2	Mini Nutritional Assessment-Short Form (MNA-SF) including an adapted version	Interview	All
Score 3	NutriMental Screener	Interview	All
Nutrition-related diseases	Self-report (e.g., diabetes, celiac disease, food allergy or intolerance)	Interview	All
Subjective evaluation of nutritional status	Single question	Interview	All
Biochemical parameters	Laboratory results, such as lipid profiles, electrolytes, liver parameters, proteins, vitamins and trace elements	CIS	Patients
Weight management	Self-report (e.g., weight changes, intentional restriction of food intake)	Interview	All
**Eating behavior (outcomes)**		
Food intake and diet quality	16 food frequency questions and additional questions regarding eating behavior	Interview	All
**Knowledge and skills (outcomes)**		
Practical nutrition knowledge	PKB-7 scale	Interview	All
Cooking and food skills		
Sum score 1	Cooking and food skills	Interview	All
Sum score 2	Cooking and food activities	Interview	All
Nutrition literacy	Single question	Interview	All
**Personality, attitudes and feelings (outcomes)**		
Personality: Consciousness	Big-Five-Inventory-short form (BFI-K)	Interview	All
Implicit theories on mental health: Incremental beliefs	Adapted scale of implicit theories on mental health	Interview	All
Feelings toward nutrition: Negative to positive	Free and spontaneous associations on nutrition	Interview	All
**Mental condition (predictors)**		
Psychiatric disorders and treatment	ICD-10 diagnoses, self-reported treatment history	Interview, CIS	Patients
Objective assessment of severity of symptoms and functioning	Health of the Nation Outcome Scales (HoNOS), m-GAF	CIS	Patients
Subjective assessment of severity of symptoms and functioning	9-item Symptom-Checklist (SCL-K-9), 9-item Patient Health Questionnaire (PHQ-9)	Interview	All
**Control measures**		
Medication	Prescribed medications and nutritional supplements	Interview, CIS	All
Personal variables	Self-reported data (gender, age, education, housing situation, source of income, physical activity, smoking status and previous nutrition counseling)	Interview	All

*CIS, Clinical information system*.

The participants' nutritional status, eating behavior and their nutrition knowledge and skills will be assessed as the primary outcomes. According to the recommendations of previous research (10), nutritional status will be evaluated based on different objective and self-reported measurements including anthropometric measurements, nutritional risk scores, nutrition-related diseases, and biochemical parameters. Eating behavior will be determined by validated food frequency questions, a diet quality index, usual diet and meal structures. Nutrition knowledge, cooking and food skills will be assessed using validated scales. As secondary outcome measures, the participants' levels of consciousness, incremental beliefs and feelings toward nutrition will be examined. The intended measurements are described below.

#### Nutrition-Related Measures

##### Anthropometric Measurements

We will measure weight (kg), height (m), abdominal girth (m) and hip girth (m), all to the nearest 0.1 units, using digital scale type SOEHNLE 63850 PWD Style Sense Compact 100 and SECA measuring tape. Each measurement will be repeated twice, and the mean-value will be assessed. Participants will be allowed to wear light clothing but no shoes. Based on these measurements, we will then calculate the body mass index (BMI; kg/m^2^) and the waist: hip ratio (WHR; abdominal girth/hip girth) to evaluate body weight and respective body fat distribution, which are both well-known cardiovascular risk factors (33).

##### Nutritional Risk Scores

As described in the introduction, there are no validated nutritional risk screening methods for people with mental disorders (9). To assess the risk for nutritional deficits, we will use three different nutrition risk scores, additional questions related to the participants' nutritional status and laboratory parameters if they were retrieved during treatment.

First, we will apply an adapted version of the Nutritional Risk Screening (NRS), which is a strong, validated and independent risk score for malnutrition in the hospital setting (10, 34). The NRS contains two components: nutritional status based on weight loss/BMI (0–3 points) and severity of disease based on a categorization of physical illnesses (0–3 points). For the latter component, we will use the modified Global Assessment of Functioning Scale (m-GAF) to evaluate the severity of psychiatric symptoms (35). M-GAF scores range from 1 to 100 with higher scores indicating better functioning: m-GAF of 91–100 means = no impairment; m-GAF of 61–90 means mild to minimal impairment; m-GAF of 41–60 means moderate to serious impairment; and m-GAF of 01–40 means pervasive impairment. A sum score of 3 points or higher indicates a risk for malnutrition.

Second, we will apply the Mini Nutritional Assessment-Short Form (MNA-SF), which is another validated and well-established screening tool for malnutrition used in hospitals and care homes (36, 37). It considers different aspects, such as decrease in appetite, problems with swallowing and digestion, BMI, weight loss, mobility, psychological stress and mental conditions. Aside from the original version, we will also use a modified version with relevant adaptations for people with SMI:

(a) We will also ask for an increase in food intake due to changes in appetite, swallowing, digestion, etc.
(b) We will also ask for weight gain.
(c) We will ask for physical mobility in general, not only limited to physical problems.
(d) We will replace questions about stress and mental condition with m-GAF ratings (see above).
(e) We will use BMI categorization based on guidelines related to obesity.

A sum score of 8 points or higher on the original MNA-SF indicates a risk for malnutrition.

Third, we will also include the NutriMental Screener, which is a nutrition screening tool in development, specifically for the mental health setting [for further information see (11)].

##### Nutrition-Related Diseases

We will ask the participants whether they have (diagnosed) nutrition-related diseases, such as diabetes, celiac disease, food allergy or intolerance.

##### Subjective Evaluation of Nutritional Status

We will ask the participants to evaluate their nutritional status (supply of energy and nutrients) on a scale ranging from 1 = very poor to 10 = very good.

##### Biochemical Parameters

For the patient samples, if available, we will collect the routinely assessed laboratory results at the patient's admission to the hospital. For example, we will acquire their lipid profiles, electrolytes, liver parameters, proteins (e.g., prealbumin and ferritin), vitamins (e.g., C, D, E, K, B1, B6, B12, and folic acid) and trace elements (e.g., zinc, iron, and selenium).

##### Weight Management

We will ask the participants whether they had lost or gained weight (in kg) in the last 3–6 months. The participants will also be asked whether they intentionally restricted food intake to regulate weight or health and to evaluate their body weight on a scale ranging from 1 = too low to 10 = too much weight (5 = just right).

##### Medication

For the clinical samples, we will acquire information on the participants' prescribed medications from the clinical information system and ask the participants whether they take the medication as prescribed and whether they take additional medications. Medication intake in the group of healthy participants (control group) will be assessed by self-reports.

In addition, we will ask the participants whether they take nutritional supplements, such as vitamin D3 or calcium. If they do, we will ask them to list the sort of supplements they are taking.

##### Food Intake and Diet Quality

Currently, there are no validated dietary assessment methods for specific use in people with SMI (4).

Comprehensive methods for assessing dietary intake, such as retrospective dietary records over several days or weighed food protocols, are often less feasible in people with SMI due to cognitive impairments, such as poor memory (38). Therefore, we will use 16 simple food frequency questions, which had been previously validated for the general Swiss population (39, 40). For instance, we will ask the participants how often they eat vegetables (daily, 4-6x/week, 1-3x/week, 1-3x/month, seldom) and how many portions they eat (1-6 handfuls). Based on these items, we will calculate a diet quality score as an indicator of a more or less healthy/ unhealthy diet (39).

##### Additional Questions Regarding Eating Behavior

The participants will be asked to state their usual number and types of daily meals (e.g., breakfast, morning snack, lunch, afternoon snack, dinner and other snacks between meals) and their usual diet (e.g., with meat, vegetarian or gluten-free). We will also ask the participants to rate their financial situations regarding their nutritional needs (very limited to an acceptable budget). In addition, the participants will rate the following questions, specifically developed for the current study, on a scale from 1 = not at all to 10 = fully agree:

- Do you enjoy cooking and eating?
- How important is a healthy and balanced diet to you?
- How important is a healthy and balanced diet to your mental health?
- Did/do your mental health conditions/psychiatric symptoms influence your eating behavior? (only for patient samples).
- Do you think that eating behavior / diet should be routinely assessed for every psychiatric/psychological treatment?
- Did you have enough time/ opportunities to discuss the relationship between nutrition and health during your previous psychiatric treatment? (only for patient samples).

Have/are the restrictions due to the COVID-19 pandemic influenced/influencing your eating behavior, and if so, in what respects?

##### Nutrition Knowledge

Practical nutrition knowledge about balanced meals will be measured using the PKB-7 scale, which had been validated for the Swiss population. The scale consists of seven items with different levels of difficulty (40). This scale aims to measure the relevant knowledge for making healthy and balanced food choices, and it has been applied in several studies and in different languages (39, 41, 42).

##### Cooking and Food Skills

To assess participants' food and cooking skills, we will use Lavelle et al. (43) newly developed and validated questionnaire. We will use a German version, which has been forward and backward translated by two different bilingual health professionals of our research team and is currently validated for the Swiss population. The questionnaire includes 14 items related to cooking skills (e.g., “Prepare and cook raw meat/poultry”) and 19 items related to food skills (e.g., “plan how much food to buy?”) rated on a scale from very poor to very good with an additional option of “never/ rarely do it.” For both scales, the scores of the cooking skill items and the scores of the food skills items will be summed up.

In addition to these newly developed items, we will also use established questions about cooking and food activities from the Social Functioning Scale (44), which had been specifically developed and validated for use in people with SMI. First, the participants will be asked to indicate how often they perform a certain activity, such as buying food or household planning (never to often). Second, they will be asked to indicate how confident they are in relation to the corresponding activities (not capable to adequate). Again, the scores will be summed up.

##### Nutrition Literacy

Aside from knowledge about nutrition or cooking and food skills as components of nutrition literacy (20), we will ask the participants how confident they feel about understanding nutrition-related health information such as notes on food packages on a scale ranging from 1 = very bad to 10 = very good. The question has been specifically developed for the current study.

##### Feelings Toward Nutrition

We will assess the participants' feelings toward nutrition using the technique of free and spontaneous associations (45) for the words “nutrition” or “diet” in a two-step approach as it has been applied in a previous study in Switzerland related to nutrition (46). First, the participants will be asked to indicate the first three words or images that come to mind spontaneously when reflecting on the words nutrition or diet. Second, the participants will be asked to value each of these associations on a scale ranging from 1 (very negative) to 7 (very positive). We will calculate the mean of the ratings as an indicator of the participants' feelings toward nutrition.

#### Mental Health-Related Measures

To evaluate the participants' mental states, a range of different measures will be used, including both objective and subjective measures.

##### Psychiatric Disorders and Treatment (Only Patient Samples)

We will assess the primary psychiatric diagnoses according to ICD-10, the existence of secondary psychiatric and somatic comorbidities, the number of previous hospital stays, treatment durations (for inpatient sample) and complications from the participants' routine medical data.

We will ask the participants how old they were when psychiatric problems occurred for the first time. In addition, participants will be asked whether they have been treated in inpatient or outpatient settings during the last 3–6 months due to poor mental or physical health conditions. Furthermore, the participants from the outpatient sample will be asked (only in the second interview) how many therapy sessions they attend per week.

##### Objective Assessment of Severity of Symptoms and Functioning (Only Patient Samples)

We will use the validated Health of the Nation Outcome Scales (HoNOS) to measure the health and social functioning (47) and the modified Global Assessment of Functioning Scale (m-GAF) to measure the severity of psychiatric symptoms (35). HoNOS and m-GAF will be measured by treating psychologists or psychiatrists during routine medical assessments upon patients' admission to the hospital. In addition, m-GAF scores will also be assessed during the interviews as part of the nutritional risk screening. The HoNOS range from 1 to 48 with higher scores indicating worse health, and the GAF ranges from 1 to 100 with higher scores indicating better functioning.

##### Subjective Assessment of Severity of Symptoms and Functioning

We will use the 9-item Symptom-Checklist (SCL-K-9), a reliable and validated short form of the Symptom-Checklist SCL-90-Revised, to assess the participants' mental states in the clinical sample (48). The SCL-K-9 is a suitable and efficient instrument for screening psychopathologic symptomatology from the last 7 days and assesses mental and physical health problems on a 5-point Likert scale. The sum score ranges from 0 to 36 with higher scores indicating more serious symptoms.

In addition, we will apply the 9-item Patient Health Questionnaire (PHQ-9) (49, 50), which is a reliable and valid screening tool for depression severity in the last 2 weeks in the medical setting. The PHQ-9 is the depression module of the PHQ for common mental disorders. The scale ranges from 0 = not at all to 3 = nearly every day, with a sum score of 10 points or higher indicating depression.

##### Personality

To assess the five personality factors (extraversion, agreeableness, conscientiousness, neuroticism and openness to experience) of the participants, we will use the validated short form (BFI-K) of the Big-Five-Inventory (BFI) (51). The participants will rate 21 statements on a Likert-type scale from 1 = fully disagree to 5 = fully agree.

##### Implicit Theories on Mental Health

The degree of participants' incremental beliefs toward their mental health (“mental health is stable vs. changeable”) will be assessed using an adapted scale by Schreiber et al. (27). The scale encompasses six items (e.g., “you can substantially change your own mental health”) ranging from 1 = strongly disagree to 7 = strongly agree.

#### Personal Variables

We will ask the participants to indicate their gender, age, education, housing situation (alone, together with others or residential care homes), source of income, the number of days in a general week in which they engage in at least 30 min of moderate-to-vigorous physical activity (52), smoking status (pack years), and consultation of a previous nutrition counseling.

#### Sample Size Calculation

Our target sample size includes a total of 192 participants, with 64 inpatients, 64 outpatients and 64 healthy adults from the general population (control group).

We calculated the required sample size for each group using G^*^Power3.1 for Windows (53) with alpha = 0.05, power 1-beta = 0.80 and medium effect size f = 0.25 based on univariate analysis of covariance (ANCOVA) regarding the outcome variable nutrition knowledge including 2 covariates (education, previous nutrition counseling). This resulted in an overall sample size of 196 participants. To obtain comparable groups, we will use stratified samples in relation to gender and age (16 female and 16 male participants between 18 and 40 years and 16 female and 16 male participants between 41 and 65 years per sample), resulting in 64 participants of each group.

#### Data Management and Analyses

The interview data will be recorded online as encoded data in LimeSurvey (32) using an individualized identification code. Data access will be password protected for the core research team only. Interview data and medical routine data will be merged in SPSS using the identification code. Data will be analyzed using SPSS and/or R. For undirected hypothesis testing, the significance level will be two-sided with α = 0.05 and for direct hypothesis testing α = 0.025.

The main statistical analyses include:

(A) Descriptive analyses of the patients' nutritional needs and problems.
(B) Comparison of samples regarding different outcome measures.
  Nutritional status (at least BMI, WHR and an adapted nutritional risk score), eating behavior (food frequency items, diet quality) and practical nutrition knowledge and skills will be compared across the three groups (inpatients, outpatients, healthy controls) using ANOVA / ANCOVA. In addition, levels of consciousness, incremental beliefs and feelings toward nutrition will also be compared. If indicated, adjustments for multiple testing will be applied (e.g., Bonferroni correction).
(C) Examination of different predictors for healthy eating such as nutrition knowledge and skills, attitudes, feelings and personality factors in psychiatric patients using regression analysis.
(D) Analysis of longitudinal associations between the course of mental condition and eating behavior in the outpatient sample using correlation analysis of two measurement points.

Missing data might occur due to refused items in the interview, the use of medical routine data, and if psychiatric treatment ends before the second interview in outpatients. The raw dataset will be analyzed for missing data patterns in terms of randomness and appropriate imputation methods (e.g., multiple or mean imputation) will be applied if necessary. For pre-post analyses in the outpatient sample, only complete datasets will be used.

## Discussion

Mental health problems are an increasing concern in public health. People with mental disorders often have poor physical health conditions, including obesity, diabetes and cardiovascular diseases (2). It is well accepted that, among others, lifestyle factors, such as inactivity, poor diet and smoking, are modifiable behavioral risk factors for physical comorbidities. Therefore, nutritional interventions in psychiatry might be promising for improving the health and quality of life of people with SMI (6). However, to date, the nutritional status and needs of people with SMI received little attention in psychiatric treatment compared to the well-established standardized assessments of malnutrition in somatic hospital patients.

Our study will provide insights into the nutritional status and eating behaviors of people with SMI and especially into possible barriers to healthy eating in this population, such as low levels of nutrition knowledge and skills, personality factors or attitudes and feelings toward nutrition. Studies on these factors have been scarce in people with mental disorders. In contrast to established risk factors, such as the unavailability of healthy food, limited financial resources, side effects of psychotropic medication and low social support, most of the examined possible barriers in our study are modifiable risk factors that can be improved through nutritional interventions.

In this study, a range of objective measures and self-reported measures will be combined and examined across different treatment settings (inpatient and outpatient samples) and longitudinally between two measurement points in the outpatient sample. Furthermore, the results of the patient groups will be controlled by the inclusion of a healthy sample. The study design should allow us to draw conclusions regarding the special nutritional needs of people with SMI in comparison to healthy people. Further, the results can help to define recommendations for nutritional risk screening in psychiatric patients and for planning effective nutritional interventions.

We carefully selected the measurements in terms of feasibility and acceptability of psychiatric patients and, therefore, also chose short scales when these were available. Although these short versions have been validated and recommended for research, their shortness might negatively affect the accuracy of the measurements, which might be a limitation of this study. To control for possible confounders in terms of the participants' eating behaviors, we will assess a range of control variables, such as personal variables and illness-related variables. Based on our pretests and previous experiences, the interviews should, on average, not take longer than 1.5 h. However, if a participant needs more time or wants to split the interview in two parts, this would be possible. Another limitation might be that our study design only allows to include psychiatric patients with primary diagnosis of F2 or F3 according to the ICD-10. Therefore, the generalizability of the results might be limited.

## Ethics Statement

The studies involving human participants were reviewed and approved by Swiss Ethics Committee, Switzerland (2019-01485). The patients/participants provided their written informed consent to participate in this study.

## Author Contributions

SM and FH are the principal investigators. The study will be conducted by LR, SM, and FH. All authors have read and approved the final manuscript.

## Funding

This research was financially supported by the Swiss Foundation for Nutrition Research (SFEFS) (grant number: 551_Rev). The SFEFS was not involved in the chosen study design, data collection, analysis or interpretation of the data, preparation, and review and approval of the manuscript.

## Conflict of Interest

The authors declare that the research was conducted in the absence of any commercial or financial relationships that could be construed as a potential conflict of interest.

## Publisher's Note

All claims expressed in this article are solely those of the authors and do not necessarily represent those of their affiliated organizations, or those of the publisher, the editors and the reviewers. Any product that may be evaluated in this article, or claim that may be made by its manufacturer, is not guaranteed or endorsed by the publisher.

## Abbreviations

SMI: Severe mental illness
PUK: University Hospital of Psychiatry Zurich
BMI: Body mass index
WHR: Waist: hip ratio
NRS: Nutritional Risk Screening
m-GAF: Modified Global Assessment of Functioning Scale
MNA-SF: Mini Nutritional Assessment-Short Form
HoNOS: Health of the Nation Outcome Scales
SCL-K-9: 9-item Symptom-Checklist
PHQ-9: 9-item Patient Health Questionnaire
BFI: Big-Five-Inventory
BFI-K: Big-Five-Inventory-short form.
